# Structure-Guided Prediction of the Functional Impact of DCLK1 Mutations on Tumorigenesis

**DOI:** 10.3390/biomedicines11030990

**Published:** 2023-03-22

**Authors:** Annalisa L. E. Carli, Joshua M. Hardy, Hanadi Hoblos, Matthias Ernst, Isabelle S. Lucet, Michael Buchert

**Affiliations:** 1 Cancer Inflammation Laboratory, Olivia Newton-John Cancer Research Institute, Heidelberg, VIC 3084, Australia; 2 School of Cancer Medicine, La Trobe University, Bundoora, VIC 3086, Australia; 3 ACRF Chemical Biology Division, Walter and Eliza Hall Institute of Medical Research, Parkville, VIC 3052, Australia; 4 Department of Medical Biology, University of Melbourne, Parkville, VIC 3010, Australia

**Keywords:** DCLK1, DCX, crystal structure, cryo-EM, missense mutations, cancer, kinase, microtubules, doublecortin domain, PEST domain

## Abstract

Doublecortin-like kinase 1 (DCLK1) is a functional serine/threonine (S/T)-kinase and a member of the doublecortin family of proteins which are characterized by their ability to bind to microtubules (MTs). DCLK1 is a proposed cancer driver gene, and its upregulation is associated with poor overall survival in several solid cancer types. However, how DCLK1 associates with MTs and how its kinase function contributes to pro-tumorigenic processes is poorly understood. This review builds on structural models to propose not only the specific functions of the domains but also attempts to predict the impact of individual somatic missense mutations on DCLK1 functions. Somatic missense mutations in DCLK1 are most frequently located within the N-terminal MT binding region and likely impact on the ability of DCLK1 to bind to αβ-tubulin and to polymerize and stabilize MTs. Moreover, the MT binding affinity of DCLK1 is negatively regulated by its auto-phosphorylation, and therefore mutations that affect kinase activity are predicted to indirectly alter MT dynamics. The emerging picture portrays DCLK1 as an MT-associated protein whose interactions with tubulin heterodimers and MTs are tightly controlled processes which, when disrupted, may confer pro-tumorigenic properties.

## 1. Introduction

Doublecortin-like kinase 1 (DCLK1) is a bi-functional protein and a member of the doublecortin family of non-classical microtubule (MT)-associated proteins (MAPs) as well as the Serine/Threonine (S/T)-kinase family [1]. As an MAP, DCLK1 is involved in MT formation and stabilization [2] and as such is potentially involved in the regulation of cellular shape and polarity, cell migration, division, and vesicular transport [3,4]. Genetic mutations of doublecortin (*DCX*) a *DCLK1* homologue, were identified as the cause of cerebral cortical malformations, such as subcortical band heterotopia (double cortex) and lissencephaly [5,6,7,8]. In contrast, DCLK1 mutations and overexpression have been associated with neurodevelopmental and neuropsychiatric disorders, including attention deficit hyperactivity disorder (ADHD), schizophrenia and bipolar disorder [9,10]. Individual knockouts of *DCX* or *DCLK1* result in moderate brain development defects, without changes in growth-cone morphology or MT distribution. However, the *DCLK1/DCX* double gene knockout in mice led to abnormal hippocampal formations and perinatal lethality, suggesting that either molecule can compensate for the absence of the other in single knockout experiments [11,12,13,14,15,16]. More specific cellular functions for both DCLK1 and DCX include neuronal migration, axon outgrowth, retrograde transport of glucocorticoid receptors, guidance of intracellular transport vesicles and mitotic spindle formation in neuroblasts [8,17,18,19,20,21,22,23].

An increasing number of reports have indicated a compelling role for DCLK1 in tumorigenesis. Two previous meta-analyses across multiple studies and cancer types showed that DCLK1 overexpression correlates with advanced clinical stage, lymph node metastasis, poorly differentiated cancers, poor overall survival, and reduced anti-tumor immune responses within the tumor microenvironment [24,25]. In addition, whole-genome sequencing of 100 matched gastric cancer pairs identified DCLK1 as a potential new driver of gastric cancer [26]. Foregoing reports have demonstrated a direct functional role for DCLK1 in promoting cancer cell migration and invasion of gastrointestinal (GI) cancers [27,28,29,30,31,32,33,34,35,36,37,38,39,40,41,42,43,44,45]. A common thread throughout those reports is that elevated expression of DCLK1 promotes pluripotency of cancer stem cells and epithelial-to-mesenchymal transition (EMT) in a kinase-dependent manner [27,28,29,30,31,32,33,34,35,36,37,38,39,40,41,42,43,44,45,46]. Accordingly, treatment with a selective small molecule DCLK1 kinase inhibitor (DCLK1-IN-1) inhibits DCLK1-dependent cell growth, migration and stemness in cancer cells [46,47,48,49,50].

Several reviews have provided in-depth discussions of the consequence of DCLK1 overexpression to cancer progression, including the functional implications of different DCLK1 protein isoforms generated from the same gene, as well as the effects of therapeutic or genetic inhibition. Although some *DCLK1* missense mutations were shown to impact protein function and stability [51,52,53,54], no specific cancer driver mutation in DCLK1 has been identified or functionally validated. Thus, this review aimed to provide a comprehensive overview of DCLK1 isoforms, the specific functions of the individual DCLK1 domains, and to predict the pro-tumorigenic functions from the most frequent somatic *DCLK1* missense mutations in humans.

## 2. DCLK1 Protein Isoforms

**Figure 1:**
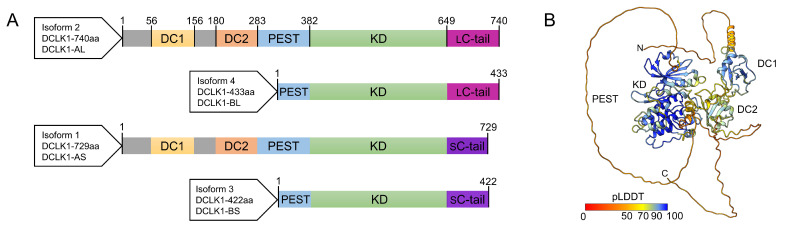
Doublecortin-like kinase 1 (DCLK1) structure and domain organization: (**A**) schematic overview of the four DCLK1 isoforms and domain boundaries comprising the two tandem doublecortin (DC) domains (yellow/orange), the proline, glutamic acid, serine, and threonine rich (PEST) linker-region (light blue), the S/T-kinase domain (KD, light green), and the short (S, purple) and long (L, magenta) regulatory C-terminal tail (C-tail); and (**B**) cartoon representation of the AlphaFold2 model of DCLK1 (Isoform 2, O15075) colored according to confidence (Predicted Local Distance Difference Test—pLDDT) (AlphaFold2 [63,64]). Regions with a pLDDT value of < 50 are likely disordered and the positions of corresponding amino acids relative to domains are uncertain. Structure rendered in UCSF ChimeraX v1.5 [65].

DCLK1 expression in humans is highly complex and is regulated via alternative promoter usage, alternative splicing, and/or post-translational cleavage, resulting in four different DCLK1 protein isoforms. The two full-length DCLK1 isoforms are presumed to be transcriptionally regulated by the 5′α-promoter where subsequent alternative splicing results in two transcripts encoding proteins that differ in the length of their respective C-terminal regulatory tail (C-tail) and are referred to as DCLK1-AS (isoform 1, 729 amino acids (aa)) and DCLK1-AL (isoform 2, 740 aa) (Figure 1A,B). Both isoforms contain four functional domains/regions: two N-terminal in tandem doublecortin (DC) domains which are responsible for the MAP function; a highly phosphorylated proline (P), glutamic acid (E), serine (S) and threonine (T) (PEST) rich linker; a S/T kinase domain (KD); and a C-tail acting as an auto-inhibitory domain (AID) that restricts kinase activity by occluding the adenosine triphosphate (ATP) binding site (Figure 1A). In certain colorectal, pancreatic, gastric, and lung cancers, this 5′α-promoter has been shown to be hypermethylated, resulting in epigenetic silencing [55,56,57,58,59] and transcriptional upregulation of the two alternative short isoforms (DCLK1-BL (isoform 4, 433 aa), DCLK1-BS (isoform 3, 422 aa)) via the alternative 3′β-promoter in intron V, which is activated by the transcription factor NF-κB [58]. These two short-protein isoforms lack the in tandem DC domains and some N-terminal residues of the PEST linker region (Figure 1A) [60,61]. However, it has been proposed that these two short isoforms may also result from calpain-mediated in vitro cleavage within the PEST region [62]. Lastly, there are two putative DCLK1 isoforms arising from predicted protein coding transcripts (DCLK1-203 (363 aa), and DCLK1-205 (56 aa)) [60], yet no evidence for their in vivo expression in humans has been reported and hence they will not be discussed any further in this review.

## 3. Doublecortin (DC) Domains

All members of the doublecortin family contain one or two DC domains, flanked by unstructured linkers [66]. A DC domain has a ubiquitin-like fold, also known as β-grasp fold, consisting of 5 β-sheets with an α-helix between the second and third β-sheet [67]. The ubiquitin-like fold is a wide-spread fold associated with a large variety of distinct cellular functions, including regulation of protein stability, signal transduction, adapter functions involved in protein–protein interactions, and RNA–protein interactions [67]. In the case of DCX and DCLK1, these domains interact primarily with MTs although some recent reports have shown an association with actin [14,68,69,70,71,72]. Only the structure of the DC1 domain of DCLK1 has been determined to date, whereas individual structures of both DC domains of DCX have been solved (Table S1). As the DC1 and DC2 domains of DCLK1 have a high sequence identity with the DC domains of DCX (80% and 87%, respectively [1]), we have utilized the available literature, experimental structures (Table S1), and structure predictions of both proteins to evaluate functional attributions to shared domains [64].

### 3.1. Distinct Roles for the DC Domains

**Figure 2:**
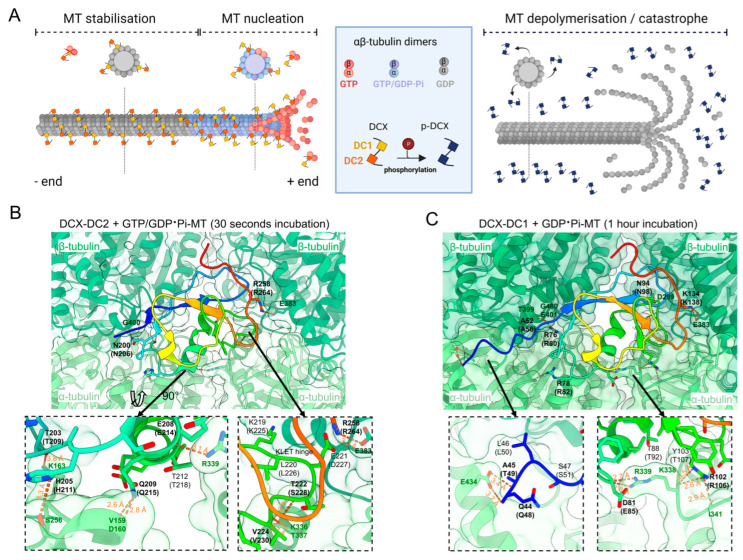
Structural basis of microtubule (MT) stabilization and polymerization by doublecortin protein (DCX): (**A**) schematic depiction of DCX domain functions. The more flexible doublecortin 2 (DC2) domain is involved in MT nucleation and preferably binds guanosine triphosphate (GTP)-αβ tubulin at the growing plus ends of the MTs. The more rigid DC1 domain binds guanosine diphosphate (GDP)-αβ tubulin and stabilizes MTs and protects them from MT catastrophe. Upon phosphorylation, both DC domains dissociate from the MTs resulting in MT depolymerization/catastrophe; and (**B**,**C**) time-resolved cryo-electron microscopy (EM) structures of DCX bound to MTs ((**B**) PDB ID: 6RF2/(**C**) PDB ID: 6REV [73]). Each DC domain is depicted in a cartoon representation (rainbow color from N- (blue) to C-terminus (red)). DCX residues which form hydrogen bonds or salt bridges with tubulin (green) are displayed as sticks and labeled in bold with the equivalent residue in DCLK1 in parentheses. Zoom panels highlight key interfaces. Distances for hydrogen bonds are indicated (orange). Schematic generated with BioRender.com and structures rendered in UCSF ChimeraX v1.5 [65].

DCX and DCLK1 bind to the lattices between the protofilaments at the corners formed by four αβ-tubulin dimers influencing MT rigidity and curvature (Figure 2) [3,73,74,75,76,77,78]. This is distinct from other classical MAPs such as TAU and MAP2 that bind to the outer ridges of the protofilaments [79]. Using functional assays, Kim et al. showed that DCX-DC1 only binds polymerized αβ-tubulin, but not unpolymerized soluble αβ-tubulin, whereas DCX-DC2 can bind to both polymerized and unpolymerized αβ-tubulin (Figure 2A) [80]. Only when expressed in tandem, but not as single domains, are DCs able to induce MT polymerization and prevent MT depolymerization, whilst isolated DC domains cannot [73,80,81]. The linker between the DC domains is predicted to be disordered, however the addition of both DCX-DC domains in trans is not able to nucleate MTs, suggesting that this connection between the DC domains has a functional role [73,74].

From biochemical and structural investigations of DCX, each DC domain appears to have a distinct role in MT nucleation and stabilization (Figure 2) [73,82]. Time-resolved cryo-EM reconstructions of DCX polymerized MTs revealed that after rapid polymerization (30 s), DC2 is bound to the four tubulin subunits (Figure 2B) [73]. However, after prolonged polymerization (1 h), DC2 is replaced by DC1 within the MT lattice (Figure 2C) [73]. DCX-DC2 appears to facilitate MT nucleation by growing plus-ends, binding to guanosine triphosphate (GTP) and the guanosine diphosphate (GDP)-dihydrogen phosphate (Pi) transition states (GDP·Pi-MTs), stabilizing tubulin-tubulin contacts in the nascent MT lattice [73]. DCX-DC2 also defines MT architecture by preferentially forming MTs with 13 protofilaments [73]. Removal of the unstructured C-terminal tail of DCX, which is highly conserved to the PEST linker in DCLK1, eliminates this preference. While this suggests that the tail is also involved in correct assembly of 13 protofilament MTs, this region has not been resolved in cryo-EM reconstructions [78]. On the other hand, DC1 is likely to favor the binding to mature lattices, including the binding to GDP-MTs, thereby providing MT stabilization. In addition, within the MT lattice, DC1 is more “rigid” and forms 11 ionic interactions from nine individual amino acids compared to the more flexible DC2 domain with only nine ionic interactions from eight amino acids (Figure 2B,C, Table S2), resulting in a surface area interaction of 1603 Å compared with 1047 Å for DC2 [73].

### 3.2. Phosphorylation of DC Domains

**Figure 3:**
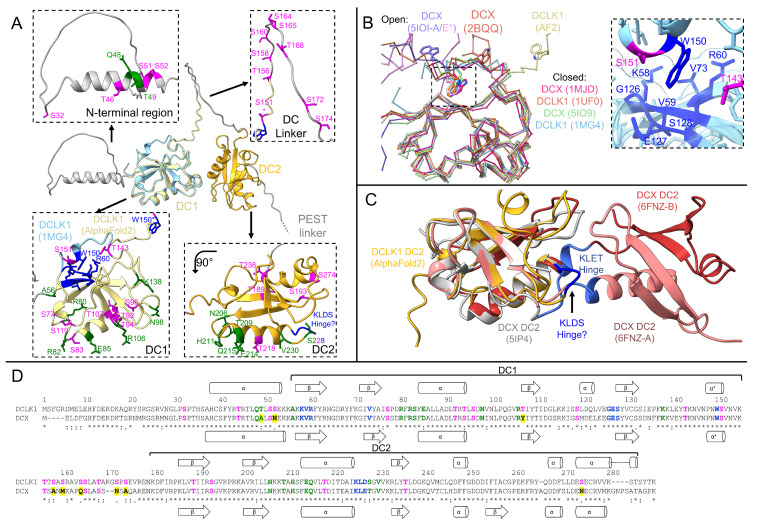
Mapping of residue function on the DC domains of DCLK1: (**A**) cartoon representations of the AlphaFold2 prediction of DCLK1 (Isoform 2, yellow/gray) and the crystal structure of DCLK1-DC1 (PDB ID: 1MG4, light blue). Zoom panels highlight functional residues in each domain: αβ-tubulin binding (green), conformational switch (blue), and auto-phosphorylation (magenta); (**B**) comparison of open and closed structures of DC1 from DCLK1 and DCX, showing only the C-alpha backbone for clarity. PDB IDs are provided in parentheses. The zoom panel shows the tryptophan (W)150 pocket in DCLK1 (PDB ID: 1MG4) with surrounding residues (blue) highlighting nearby phosphorylation sites (magenta); (**C**) comparison of monomeric structures of DC2 with the domain swapped dimer structure (salmon/red). The KLET hinge in DCX which flips out to mediate the domain swap, and the equivalent residues in DCLK1 (KLDS), are highlighted in blue; and (**D**) sequence alignment of DCLK1 and DCX colored according to assigned function as in (**A**). Differences in functional residues are highlighted in yellow. Experimentally determined and predicted secondary structures are depicted. Residue numbers for DCLK1 are shown above with the boundaries of the DC domains indicated. Sequence alignment performed using PROMALS3D [84] and structures rendered in UCSF ChimeraX v1.5 [65].

While phosphorylation can be involved in forming recognition sites for interactors, phosphorylation of the DC domains, N-terminal region, and DC1-DC2 linker also negatively impact tubulin binding (Figure 3 and Figure S1). Two in vitro studies have shown that unphosphorylated DCLK1 has a higher tubulin polymerization rate than phosphorylated DCLK1, and that the (hyper)-phosphorylated DC domains result in reduced MT binding affinity [52,53]. A total of 29 phosphorylation sites have been identified in DCLK1-DC domains: 5 in the N-terminal region; 10 in DC1; 8 in the DC linker; and 6 in DC2 (Table S2, Figure 3A,D and Figure S1). Phosphorylation sites have either been identified by mass spectrometry-based phospho-proteomics of recombinantly expressed protein or cell-wide studies. Nevertheless, the phosphorylation state of DCLK1 might differ in vivo especially in tumorigenic cells. As no high-resolution structures of DCLK1 bound to tubulin are available, structural analysis of DCX-tubulin structures is warranted to assign putative functions of conserved regions between the two proteins.

Although the AlphaFold2 algorithm predicts alpha-helical arrangements for the N-terminal region adjacent to DC1 in both DCX and DCLK1 [63,64], in the cryo-EM structure of DCX-DC1 bound to tubulin, the N-terminal region is extended along a groove between αβ-tubulin and anchored to α-tubulin through a hydrogen bond between the backbone of alanine (A)45 and glutamine (Q)44 in DCX and E434 in α-tubulin (Figure 2C). Phosphorylation of nearby S47 (S51 in DCLK1, Figure 3A) is suspected to weaken this interaction, as a phospho-mimetic of DCX (S47E) showed reduced MT binding [16,17]. The N-terminal region of DCLK1 contains two additional nearby phosphorylation sites not present in DCX, DCLK1-T49 (A45 in DCX) and DCLK1-S52 (asparagine (N)48 in DCX) that may provide additional points of regulation, these residues have been shown to be auto-phosphorylated in vitro by mass spectrometry (Figure 3D) [52,83].

From the αβ-tubulin interacting residues identified in DCX-DC1 and DCX-DC2, there are several phosphorylation sites located within the equivalent tubulin binding faces in DCLK1: S77, S83, T92, T94, S96, T107 in DC1; and T218 and S228 in DC2 (Figure 3A,D, Table S2) [20,49,52,53,83]. These S/T residues are highly conserved between DCX and DCLK1, except for T107, which corresponds to tyrosine (Y)103 in DCX (Figure 3D). The location of these phosphorylation sites in the vicinity of key functional residues for αβ-tubulin-binding region suggests a mechanism whereby phosphorylation disrupts the isomeric binding to αβ-tubulin, resulting in dissociation of DCLK1 from MTs.

Like the disordered PEST domain (discussed below), the N-terminal and DC-linker regions are predicted to be hot-spots for protein–protein interactions (Figure S1A,B). Interactors identified by in silico prediction analysis include proteins involved in cell-cycle processes, protein degradation, and the MAPK and Wnt signaling cascades. In addition, many auto-phosphorylation sites have been identified in these flexible disordered linker regions via mass spectrometry analysis (Figure S1A,B, Table S2) [52,53,83].

### 3.3. The Open and Closed Conformation of DC1

The first structure of DCLK1 was obtained in 2003 by Kim et al., who successfully crystallized the bacterially expressed DCLK1-DC1 domain (PDB ID: 1MG4, Figure 3A,B) [68]. That same year a nuclear magnetic resonance (NMR) structure ensemble of DCLK1-DC1 was solved by the RIKEN Structural Genomics/Proteomics Initiative (RSGI) (PDB ID: 1UF0). The core of the domain in both structures is highly similar (residues 55–153, all atom root mean square deviation (RMSD) = 1.3 Å) whereas the N- and C-termini diverge (Figure 3B).

Conformational changes within the C-terminal part of the DC1 region have been proposed to modulate the DC domain arrangement during MT binding. X-ray crystallography and NMR structures of DCLK1-DC1 and DCX-DC1 have revealed a tryptophan (DCLK1-W150, DCX-W146) that mediates the conformational change between an “open” and “closed” state (Figure 3B, Table S1). Most structures have been solved in the “closed” state, in which the tryptophan sits in a highly conserved pocket formed by lysine (K)58, valine (V)59, arginine (R)60, V73, glycine (G)126, E127, and S128 in DCLK1, and residues 54–56, 69, 122–124 in DCX, respectively (Figure 3B,D). In two structures of DCX-DC1, an NMR structure (PDB ID: 2BQQ) and a crystal structure (PDB ID: 5IOI, chains A and E) the tryptophan is flipped out into a so called “open” conformation (Figure 3B and Table S1). Interestingly, the “open” conformation of the crystal structure is stabilized by a small domain swap where the T146 from one chain binds into the tryptophan pocket of the other chain, but whether this is a conformational switch mechanism, or an artifact of crystallization is unclear (Figure 3B). Although AlphaFold2 predictions of DCLK1 show an “open” conformation (Figure 3B), the confidence of this region is very low (pLDDT < 50). If the DC-linker was fully extended, a switch to the “closed” conformation would shorten the distance between DC1 and DC2 domains in DCX by approximately 20 Å [85]. It is possible that the nearby residues T143 and S151 (Figure 3A,B), which have been shown to be phosphorylated in vitro [52,53,83], may have a role in modulating this conformational switch. As the cryo-EM reconstructions of DCX bound to tubulin lack density for this region [73], it is unknown whether this conformational change is involved in MT binding.

### 3.4. Domain Swap of DC2

The DC2 domains of DCLK1 and DCX have been more challenging to study by X-ray crystallography and NMR. Multiple studies have demonstrated the thermodynamic unstable nature of the isolated DC2 domains of DCLK1 and DCX, resulting in partially unfolded proteins and protein aggregates [73,75,80]. Nevertheless in 2016, Burger et al. were able to crystallize DCX-DC2 in complex with a camelid antibody fragment (PDB ID: 5IP4, Figure 3C) [75]. They also obtained diffracting crystals of DCX-DC2 alone which were difficult to phase [75], but they later published the solution in 2018 of the first and only structure of a domain swapped dimer of DCX-DC2, in which half of a DC domain flips out to complete a DC domain on a second molecule (Figure 3C) [86]. The hinge which mediates the domain swap (KLET, 219–222), is partially conserved in DCLK1 (KLDS, 225–228) and may be able to function in an analogous manner (Figure 3C,D). If DCLK1 was able to dimerize in this manner in vivo, this would have important implications for oligomerization and stabilization of MTs. The cryo-EM structure of DCX-DC2 bound to MTs (PDB ID: 6RF2) is monomeric but differs from the monomeric crystal structure especially at the tubulin interface (all atom RMSD = 2.8 Å), and an additional 11 residues were resolved at the C-terminus (residues 254–264) [73].

### 3.5. F-Actin Binding of DC Domains

Several residues in the N-terminal region preceding the DC domains of DCX are confirmed substrates of multiple kinases and phosphatases; DCX-S47 is phosphorylated by MT affinity-regulating kinase 1 (MARK1) and protein kinase A (PKA) [20,74], while the corresponding S51 site in DCLK1 has been identified as an auto-phosphorylation site [83]. Using a phospho-mimetic S47E substitution in DCX, two independent studies observed a reduction in MT binding and an increase in F-actin binding, resulting in filamentous actin and lamellipodia formation accompanied by increased cell migration [68,87]. DCX has been shown to interact with known actin binding/regulating proteins such as Neurabin II which binds F-actin [68,88] and spermatogenesis associated 13 (SPATA13) which is known to activate Rac family small GTPase 1 (RAC1) leading to lamellipodia formation [87].

DCX-S28 is a substrate of cyclin-dependent kinase 5 (CDK5) [70,89] and acts as a switch between MT binding in its unphosphorylated state, and F-actin binding when phosphorylated [70]. Since DCX-S28 is a conserved residue and aligns with DCLK1-S32 (Figure 3A,D), we hypothesize that this switch is conserved between DCX and DCLK1, especially as phosphorylation of S32 in DCLK1 has been identified in 22 phospho-proteomic data-sets [90]. Based on the above, we speculate that phosphorylation of key residues in the N-terminal region of DCLK1 may act as a molecular switch conferring the ability to regulate cytoskeletal dynamics at both the microtubular and actin filament level.

## 4. PEST Linker Region

The function of the PEST linker region (residues 283–381) in DCLK1 is poorly understood and it is predicted to be disordered according to AlphaFold2 (Figure 1B). PEST sequences are present in many transcription factors, kinases, and cell-cycle regulators with a high turnover and are known to act as versatile contact sites for protein–protein interactions [87,88,89,90] and targeted degradation [91,92,93,94]. In silico prediction analysis of the PEST region identified 35 binding motifs for 30 unique proteins (Figure S1C) [95]. These proteins are associated with cell cycle processes, the MAPK and Wnt signaling cascades, and protein degradation [96].

Depending on their specific motifs, PEST domains can be recognized by various proteolytic regulators [94,97,98,99]. CaSpredictor, a computer-based prediction tool identified that ~56% of the caspase substrate motifs are localized within PEST regions of proteins [100], supporting the predicted caspase 3/7 cleavage site (D369|G370) found within the DCLK1 PEST domain (Figure S1C). The identified calpain cleavage site (S323|T324) is supported by in vitro studies which found no difference in susceptibility to calpain for the different DCLK1 isoforms [62,101], and that cleavage is independent of phosphorylation [52]. Proteolytic cleavage has only been observed in vitro using purified DCLK1 or within neuronal cells where the cleaved KD-containing C-terminal fragment translocated into the nucleus [52,62]. It is unclear whether these events occur in vivo or in a cancer setting.

The PEST linker region of the zebrafish ortholog DCLK2, which shares a 60% sequence similarity with the human DCLK1 PEST domain, has been proposed to drive localization to MTs [102,103]. Similar observations were made in DCX, where truncation of the PEST domain resulted in reduced MT association in cells [104]. Nevertheless, these studies did not address whether phosphorylation of the PEST domain directly impacted the ability of the DC domains to interact with αβ tubulin.

Several threonine and serine residues within the PEST domains of DCX and DCLK1 are predicted substrates for a number of protein kinases including CDK5, CDK1, glycogen synthase kinase 3 (GSK3), mitogen-activated protein kinase 3 (MAPK3/ERK1) and c-Jun N-terminal kinase (JNK) (Figure S1C, Table S2) [66]. However, the functional contribution of most of these kinases remains unvalidated experimentally with the exception of CDK5 (S297), GSK3 (S297), and JNK1 (T321, T331, S334) for their phosphorylation of DCX [18,20,70,89,105,106]. Interestingly, in silico analysis of the DCLK1-PEST region predicted binding sites for GSK3, ERK1, CDK1 and CDK5 (Figure S1C). Other prominent kinases that are predicted to interact with the PEST domain are cyclic AMP dependent protein kinase A, C, and G (PKA, PKC, and PKG), aurora kinase B (AURKB), and casein kinase 1, and 2 (CK1, CK2) (Figure S1C).

In DCX, phosphorylation of S297 shifted the distribution of DCX away from MT bundles, and a phospho-mimetic S297D substitution led to impaired MT polymerization in vitro [18]. Phosphorylation of the DCX-PEST domain by JNK1 promoted its mobilization to the growth cones of the leading edge in migrating neurons, suggesting a function for these phosphorylation sites in actin cytoskeleton dynamics [105,107]. In addition, inhibition of the phosphatase PP2A resulted in a rapid loss of DCX localization to the neurite tips and DCX accumulation in the cell body [20]. Together, these studies strongly suggest a context-dependent function for the PEST linker region phosphorylation that likely drives the spatial and temporal localization of DCX and DCLK1.

## 5. Kinase Domain and C-Terminal Regulatory Tail

DCLK1 contains a functional S/T-kinase domain (residues 382–648) and a C-tail (residues 649–729 in isoform 1 and 649–740 in isoform 2). Its closest homolog is the Ca^2+^/calmodulin-dependent protein kinase 1 (CAMK) domain with 46% sequence identity [53], albeit with DCLK1 lacking the calmodulin binding domain found in CAMK family members.

**Figure 4:**
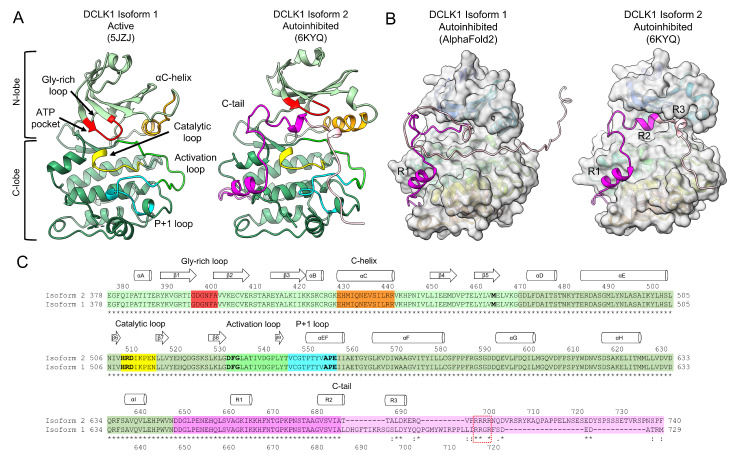
The structure and conformations of the DCLK1 kinase domain: (**A**) cartoon representation of DCLK1 structures in the active conformation (left, isoform 1, PDB ID: 5JZJ) and autoinhibited conformation (right, isoform 2, PDB ID: 6KYQ), with the N-lobe (light green) and C-lobe (olive green) indicated. Catalytic and regulatory regions are highlighted: the glycine rich loop (red), αC-helix (orange), the catalytic loop (yellow), the activation loop (green), the P + 1 loop (cyan), and the C-tail (purple/pink); (**B**) comparison of the C-tail structure predicted by AlphaFold2 [63,64] for isoform 1, and the auto-inhibited structure of isoform 2. The kinase domains are depicted in a surface representation excluding the C-tail portion. The three regulatory helices of the C-tail (R1, R2, R3) are indicated; and (**C**) sequence alignment of DCLK1 isoforms 1 and 2 colored according to assigned function as in (**A**). Key functional motifs are bolded: the gatekeeper residue (M465), HRD-motif in the catalytic loop, DFG-motif in the activation loop, and the APE-motif in the P + 1 loop. Secondary structure is shown for isoform 2. The Arginine (R) rich region in the C-tail is partially conserved (dashed box). Sequence alignment performed using PROMALS3D [84] and structures rendered in UCSF ChimeraX v1.5 [65].

The first X-ray crystal structure of DCLK1 kinase domain (residue 374–648, PDB ID: 5JZJ) showed that the kinase domain adopts a typical active-like conformation (DFG-in) stabilized by the canonical K419 and E436 salt bridge interactions, thereby orienting the αC-helix in an active conformation (Figure 4A and Table S1) [53]. A recent structure of DCLK1 isoform 2 (residues 380–701, PDB ID: 6KYQ) shows that inclusion of the C-tail does not impact on the active-like conformation but confers its auto-inhibition (Figure 4B) [51]. The C-tail of DCLK1 contains two α-helices (R1 and R2) and a 3_10_ helix (R3). In the auto-inhibited conformation, the R1 helix wraps around the C-lobe, and the R2 and R3 helices occlude the ATP-binding site cleft (Figure 4B,C) [51]. Interestingly, K692 within the 3_10_ R3 helix forms a salt bridge with the catalytic aspartic acid (D)511 of the HRD motif and D533 in the conserved DFG motif to competitively block catalytic activity [51]. An additional feature of the C-tail is an arginine-rich sequence (698–701, RRRR in isoform 2; 717–720, RRGR in isoform 1), where R698 is proposed to stabilize the C-tail to the kinase domain. Despite the different length of the C-terminal tail across the DCLK1 isoforms, most of the features of these tails remain conserved (Figure 4C), suggesting that the auto-inhibitory action of the C-tail is likely to be a conserved mechanism across all isoforms. Although an auto-inhibited structure of DCLK1 isoform 1 has yet to be determined experimentally, AlphaFold2 predicts an occluded ATP-binding site similar to that observed for isoform 2 (Figure 4B) [63,64].

Several studies have demonstrated the importance of DCLK1 auto-phosphorylation activities on its MT function. A “kinase-dead” DCLK1^D511N^ mutant has higher tubulin polymerization activities than DCLK1^WT^ [53,54]. Corroborating these findings, the addition of recombinant phosphatase to DCLK1^WT^, also increases DCLK1 tubulin polymerization activity [53,54]. Therefore, it is likely that the auto-inhibitory function of the C-tail is regulated through phosphorylation. Residue T688, localized between R2/R3 helices of the C-tail (Figure 4C), was proposed as a phosphorylation-dependent switch [52], and it is likely that phosphorylation at that site would release the auto-inhibitory activity of the C-tail. A recent study identified Hippocalcin-like 1 (HPCAL1) protein as a potential positive regulator of the DCLK1 kinase activity by releasing the C-tail through direct interaction in a Ca^2+^-dependent manner [51].

Aside from auto-phosphorylation, few DCLK1 substrates have so far been identified and functionally validated. DCLK1 phosphorylates MAP7 domain-containing protein 1 (MAP7D1) on S315, which is involved in MT cytoskeleton organization [108,109]. Another study reported S177 and S181 on inhibitor of NF-κB (IKKβ) as specific targets of DCLK1 kinase activity in macrophages of a murine arteriosclerosis model. [110]. Whether this activation is also observed in humans and cancer inflammation, has yet to be determined.

A recent phospho-proteomics study utilizing recombinant DCLK1 and dephosphorylated HeLa cell lysate revealed phosphorylation of 223 S/T/Y-sites across 164 proteins, of which 12 were identified as auto-phosphorylation sites on DCLK1 (Table S2) [83]. Ferguson et al. identified 29 significantly decreased (*p* < 0.05, Log2FC < −0.5) phosphorylation sites on 20 different proteins, mainly involved in cell motility, after treatment of human pancreatic cancer organoids with DCLK1-IN-1 [48], whereas DCLK1-IN-1 treatment in colorectal cancer cells resulted in the downregulation of 63 phosphorylation sites on 37 proteins [49]. The latter study identified CDK11, Matrin-3 (MATR3), and DNA topoisomerase 2-beta (TOP2B) as potential DCLK1 substrates [49]. CDK11 and MATR3 are both involved in RNA-processing, whereas TOP2B is involved in DNA replication and gene transcription [108]. Lastly, the short DCLK1 isoforms 3 and 4, which only contain the PEST and KD domains, are significantly upregulated in advanced and more aggressive colorectal and esophageal cancers [40,57,58,111,112,113,114].

However, the lack of overlap between the potential substrates within these studies supports the existence of context and isoform dependent DCLK1 substrates. This not only highlights that the catalytic activity of DCLK1 may regulate additional tumor-promoting cellular processes, but also argues for a need to develop (isoform) selective DCLK1 inhibitors to dissect the contribution of DCLK1 kinase activity to tumorigenesis [54].

## 6. Predicted Functional Impact of Critical DCLK1 Mutations

According to the National Cancer Institutes’ genomic data commons portal, the *DCLK1* gene is mutated in 394 out of 13,582 cases across 31 projects which include over 20 different cancer types and primary sites [115]. DCLK1 is most frequently mutated in stomach adenocarcinomas (STAD, 10.6%), uterine corpus endometrial carcinoma (UCEC, 9.2%) and colon adenocarcinomas (COAD, 8.7%), while copy number variation gains are most frequently observed in rectal adenocarcinomas (READ, 69.5%), COAD (56.3%), and STAD (37%) (Figure S2A). The frequency and location of the 229 missense mutations (58.1% of mutations) in DCLK1 are shown for each amino acid in Figure S2B, with the most frequent mutations located within the N-terminal region and the DC1 domain, suggesting that these amino acids may have functional importance and a role in oncogenesis.

Within the N-terminal region, the substitution of the hydrophobic and neutral alanine at position 18 with a slightly bulkier hydrophobic and neutral valine is observed four times (Figure S2B) but due to the similarities between the amino acids [116], the functional impact of the A18V substitution remains unclear. R45 is the most frequently (7×) mutated amino acid and is located just upstream of the first DC domain. Of the 7 cases with R45 mutations, 5 are R to cysteine (C) substitutions, the other two are R to leucine (L) and R to histidine (H) substitutions. As arginine and histidine are similar in size and charge, this substitution most likely will not affect protein function, unlike the hydrophobic leucine and cysteine which are predicted to disrupt existing salt-bridges [116]. Interestingly, phosphorylated T42 in DCX (T46 in DCLK1) is recognized by 14-3-3ε binding protein, protecting DCX from degradation [117,118]. Whether 14-3-3ε interacts with DCLK1 in a similar fashion is yet to be elucidated, however recent work by Buljan et al. explored the interaction network of 300 kinases, including DCLK1. These authors identified several 14-3-3 family members (ε, β, ζ, η, and θ) as partners in DCLK1 co-immunoprecipitation studies [119]. Thus, mutations of amino acids neighboring R45 could potentially alter protein–protein interactions and the corresponding protein stability of DCLK1. A recent study by Lu et al. has shown that the N-terminal linker region of DCX is important for facilitating kinesin motor binding along MTs. Accordingly, S47R substitution (corresponding to DCLK1-S52) results in cellular mislocalization of kinesin-3 motor proteins [70,74]. Meanwhile, the functional roles of different post-translational modifications in the disordered N-terminal regions of DCLK1 are yet to be addressed.

Substitutions in DCLK1 that are most likely to impact on MT binding are T49 to methionine (M), R80Q/W, and K138N located within DC domains, as these residues configure isomeric binding to αβ-tubulin residues described above (Figure 2C and Figure 3A,D). Especially the positive polar R80 and K138 residues (R76 and K134 in DCX, respectively), that interact with the negatively charged E401 and E383 residues of β-tubulin, respectively (Figure 2C). If DCLK1 binds tubulin by an analogous mechanism, the substitution of the arginine at position 80 by either the larger hydrophobic tryptophan or the smaller uncharged polar glutamine, is likely to disrupt the salt bridge needed for MT binding (Tables S2 and S3). A similar disruption of an ionic bond with E383 of β-tubulin would occur when K138 is substituted by the polar, uncharged asparagine. The T49M substitution changes not only the phosphorylation site, potentially impacting MT binding, but the methionine is predicted to destabilize the interaction with α-tubulin due to its substantially larger size and hydrophobic nature (Table S3) [116].

Due to their spatial proximity to W150, the V59A and R60C substitutions are likely to impact on the open-close DC conformation (Figure 3A–C) as they result in a loss of hydrophobic interactions and thus are predicted to destabilize the open conformation (Table S3). If the hinge which mediates the DCX-DC2 domain swap (KLET, 219–222, [86]) functions similarly in DCLK1-DC2 (KLDS, 225–228), the substitution K225T may have an impact on the conformational landscape of this domain.

Multiple (auto-)phosphorylation sites within the DC linker and PEST region have been identified for both DCLK1 and DCX and have been functionally linked to altered localization and MT polymerization activity [18,20,66,70,89,105,106,107]. Accordingly, substitutions of any of the phosphorylated serine or threonine residues (S158W, S305G, T311I, S330L, S334L, and S337N) are anticipated to result in functional changes (Figure S1C). In the DC2 domain, T236 has been identified as a specific auto-phosphorylation site for the full-length DCLK1 compared to C-tail-deleted DCLK1 protein isoforms; indeed Agulto et al. have suggested similar consequences arising from the T688A mutation [52]. The relatively high frequency of substitutions (T236A/M) at this residue suggests that this threonine may have functional importance (Figure S2B).

Amino-acid substitutions in the PEST region could also affect proteolytic cleavage. The frequent R326H/C substitution (Figure S2B) is predicted to disrupt calpain recognition and cleavage according to the deep learning model of calpain cleavage sites [120] and may lead to the loss of the CDK5 recognition motif to phosphorylate T324 according to Scansite v4.0 [95]. The mutation N368T modifies the predicted canonical recognition motif for Caspase 3/7 and increases the probability of cleavage according to the SitePrediction web server [121].

Most mutations within the kinase domain have been classified as either altering functional or structural integrity and therefore having a negative impact on the catalytic activity [53]. Substitutions most likely affecting catalytic activity are located within the glycine-rich loop (N400H), αC-helix (E430K, V443L), and the activation loop (G542V/D, P556A) which includes the DFG motif (G535E), and HRD motif (D511N, kinase-dead mutant) (Figure 4C). The remaining substitutions identified within the N- or C-terminal lobes of the KD are predicted to cripple structural integrity (Table S3) [53]. Additionally, mutations identified within the C-tail are predicted to result in conformational changes that alter kinase activity as outlined above. Substitutions within the R1 helix (K664N) and kinase hinge region (I477T) are predicted to be destabilizing (Table S3) and may result in conformational changes that could lead to either kinase activation or inactivation. Substitutions within the R2 helix (S683Y, A686T) are also predicted to be destabilizing (Table S3) and could disrupt the hydrophobic core within the catalytic domain, thus freeing the ATP-binding pocket [51].

## 7. Conclusions

**Figure 5:**
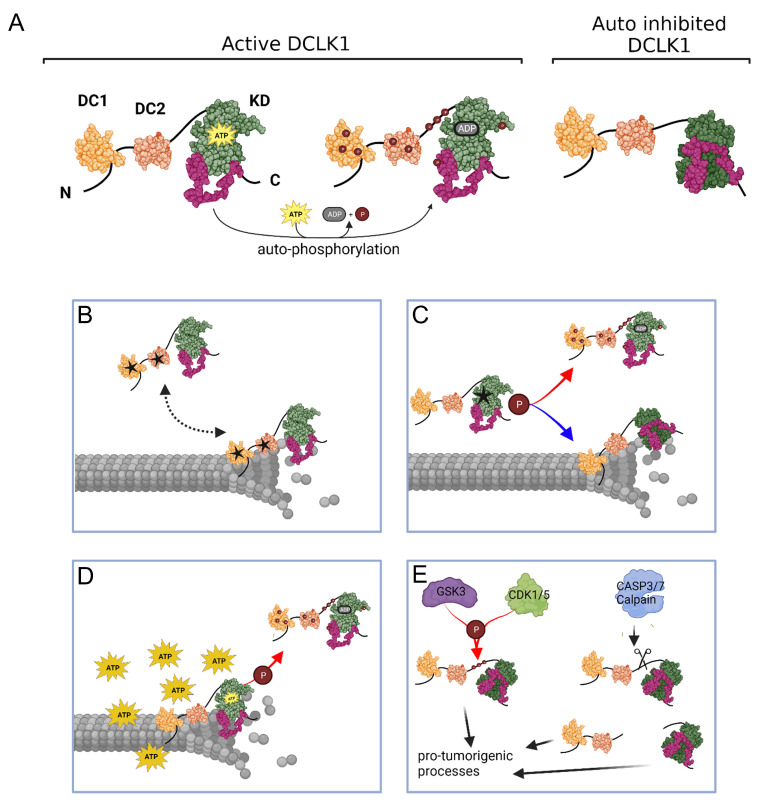
Schematic summary depicting how DCLK1 mutations can affect MT dynamics: (**A**) the active and autoinhibited form of DCLK1: (**B**) mutations within the DC domains can interfere with MT binding, affecting MT polymerization and stability; (**C**) as can mutations which increase or decrease kinase activity; (**D**) increased kinase activity can result due to excess adenosine triphosphate (ATP) in cancer cells; and (**E**) additionally, regulation by upstream kinases or cleaved products can lead to activation of pro-tumorigenic processes. Figure created with BioRender.com.

DCLK1 is a MT-associated, multidomain protein with a functional S/T-kinase domain whose auto-phosphorylation activity is required to overcome autoinhibitory interactions with its C-terminal tail (Figure 5A). The DCLK1 somatic missense mutations in solid cancers are almost evenly dispersed throughout every domain of DCLK1. Most of these mutations are predicted to impact MT binding/dynamics either: (1) by directly altering residues in the DC domains, PEST linker or the intervening sequences important for tubulin or MT binding, or (2) by altering DCLK1’s auto-phosphorylation ability which will alter its capacity to associate with MTs (Figure 5B,C). Thus, mutations within the kinase domain are more likely to inhibit kinase activity, whilst mutations in the C-tail/AID are predicted to increase kinase activity by removing the pseudo-substrate and freeing up access for ATP at the catalytic site. The latter may be of particular relevance in cancer cells, which often have an excess of cellular ATP (known as the Warburg effect) [122], and could outcompete the C-tail resulting in the hyper-phosphorylation of the DCs, PEST and linker regions and thus dissociation from MTs (Figure 5D). Moreover, the presence of numerous predicted upstream kinase phosphorylation sites [106], which are frequently disrupted by missense mutations, suggests the existence of alternative signaling pathways able to initiate pro-tumorigenic processes. In addition, the protein products resulting after proteolytic cleavage of DCLK1 by calpain and caspases 3/7 may also lead to pro-tumorigenic processes as recently demonstrated for the short isoforms of DCLK1 in colon, gastric, lung and pancreatic cancer [55,56,57,58,59].

Assuming that a majority of somatic missense mutations in DCLK1 have oncogenic potential, it is somewhat puzzling that mutations in the N-terminal half of the protein seem to reduce association with MTs while mutations in the C-terminal half seem to have the opposite effect. Although at first glance this may appear paradoxical in light of the tightly regulated DCLK1/MT interactions, it could suggest that affecting the ratio of DCLK1 bound to MTs interferes with proper MT dynamics, which underpins mitosis, cell signaling, trafficking migration and other cancer-relevant activities. Indeed, such a bimodal mechanism on MT dynamics has been demonstrated for MT depolymerase mitotic centromere-associated kinesin (MCAK) [123]. However, there may be DCLK1-dependent tumor-promoting mechanisms independent of its MT association. This is supported by studies where phosphorylation of key residues at the N-terminal region of DCX regulate the dual actin/tubulin binding activity of DCX [124]. In addition, the co-localization of phosphorylated DCX with cellular protrusions and focal adhesions in migrating cells is an actin polymerization-dependent cellular process [125]. Ultimately, to properly assess the pro-tumorigenic effects of DCLK1 mutations, protein- and cell-based studies with individual missense mutants and isoforms need to be conducted.

Many studies have shown that the overexpression of DCLK1 in cancer cells results in induction of EMT, stemness and increased cell migration and invasion, which can be reversed with small molecules targeting the kinase domain of DCLK1 [31,34,35,46,47,126]. In addition, targeting DCLK1 with anti-DCLK1 monoclonal antibody (CBT-15) or siRNAs showed promising results in reducing tumor burden, cancer stemness, invasion, and metastasis [31,35,41,46,47,48,49,50,51,54,127,128,129,130,131,132,133,134]. It remains to be shown whether such anti-DCLK1 targeting approaches are equally effective in the context of the various DCLK1 missense mutations that promote tumorigenesis.

## Data Availability

Publicly available datasets were analyzed in this study. This data can be found here: DCLK1 mutations: https://portal.gdc.cancer.gov/genes/ENSG00000077279 (open access data accessed on 10 October 2022) DCLK1 domain structure data: https://www.rcsb.org/ (uniprot ID: O15075, list of PDBIDs accessed available in Table S1). Protein FASTA formats: https://www.uniprot.org/ (uniprot IDs: O15075, O43602, accessed on 11 January 2023).

## Supplementary Materials

The following supporting information can be downloaded at: https://www.mdpi.com/article/10.3390/biomedicines11030990/s1, Figure S1: In silico prediction of proteins that interact with the (A) N-terminal region, (B) DC-linker region, and (C) PEST linker region for isoforms 1 & 2 (top) and isoforms 3 & 4 (bottom); Figure S2: DCLK1 missense mutations in cancer; Table S1: Experimentally determined structures of the DC1 and DC2 domains from DCX and DCLK1, and the DCLK1 kinase domain; Table S2: Functional residues of DCLK1 and their mutation frequency; Table S3: Predicted impact of point mutations on the structural stability of DCLK1. References [18,20,48,49,51,52,53,54,70,73,74,75,80,83,85,86,89,95,105,107,115,120,135,136,137,138] are cited in the Supplementary Materials.
