# Supplementary material for: Structure-Guided Prediction of the Functional Impact of DCLK1 Mutations on Tumorigenesis
Source: Biomedicines. 2023 Mar 22;11(3):990. doi: 10.3390/biomedicines11030990 (PMC10046695; doi:10.3390/biomedicines11030990)
Supplement: Supplementary file 1 [file biomedicines-11-00990-s001.zip › biomedicines-2281970-supplementary.pdf]

Carli *et al.* Supplementary Tables and Figures Index

Figure S1. In silico prediction of proteins binding to the (**A**) N-terminal region, (**B**) DC-linker region, and (**C**) PEST linker region for isoforms 1 & 2 (top) and isoforms 3& 4 (bottom)……………………………………2

Figure S2. DCLK1 missense mutations in cancer. (**A**) The distribution of the 394 DCLK1 mutations and CNV gains and losses identified in the GDC data-set for each malignancy. (**B**) Lollipop figure showing frequency and position of the DCLK1 missense mutations identified within the GDC cohort..…………..….3

Table S1. Experimentally determined structures of the DC1 **and** DC2 domains from DCX and DCLK1 and the DCLK1 kinase domain……………………………………………………………………………..…..4

Table S2. Functional residues of DCLK1 and their mutation frequency………………………………………..6

Table S3. Predicted impact of point mutations on the structural stability of DCLK1…………………………………11

References…………………………………………...……………………………………………………...12

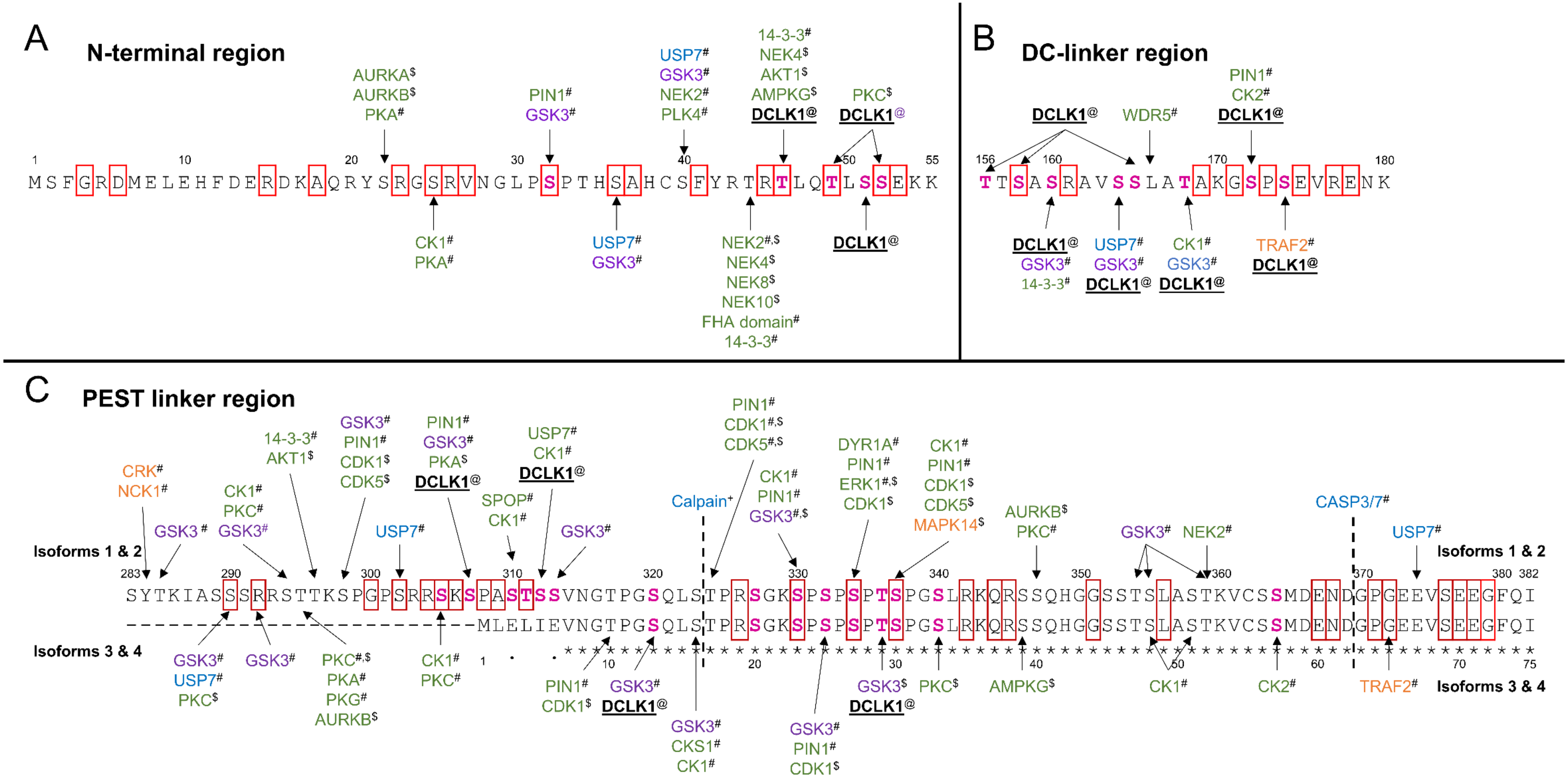

Figure S1. In silico prediction of proteins that interact with the (**A**) N-terminal region, (**B**) DC-linker region, and (**C**) PEST linker region for isoforms 1 & 2 (top) and isoforms 3 & 4 (bottom). Proteins are colored by function: protein degradation (blue), cell cycle processes (green), MAPK cascade (orange), and Wnt signalling (purple). Proteins were identified by Eukaryotic Linear Motif resource (ELM, #) [135], Scansite 4.0 ($) [95], or DeepCalpain (+) software [120], or manually curated from the literature with DCLK1 autophosphorylation sites (@), phosphorylation sites (magenta), and identified mutations (red boxes) from supplementary Table S2.

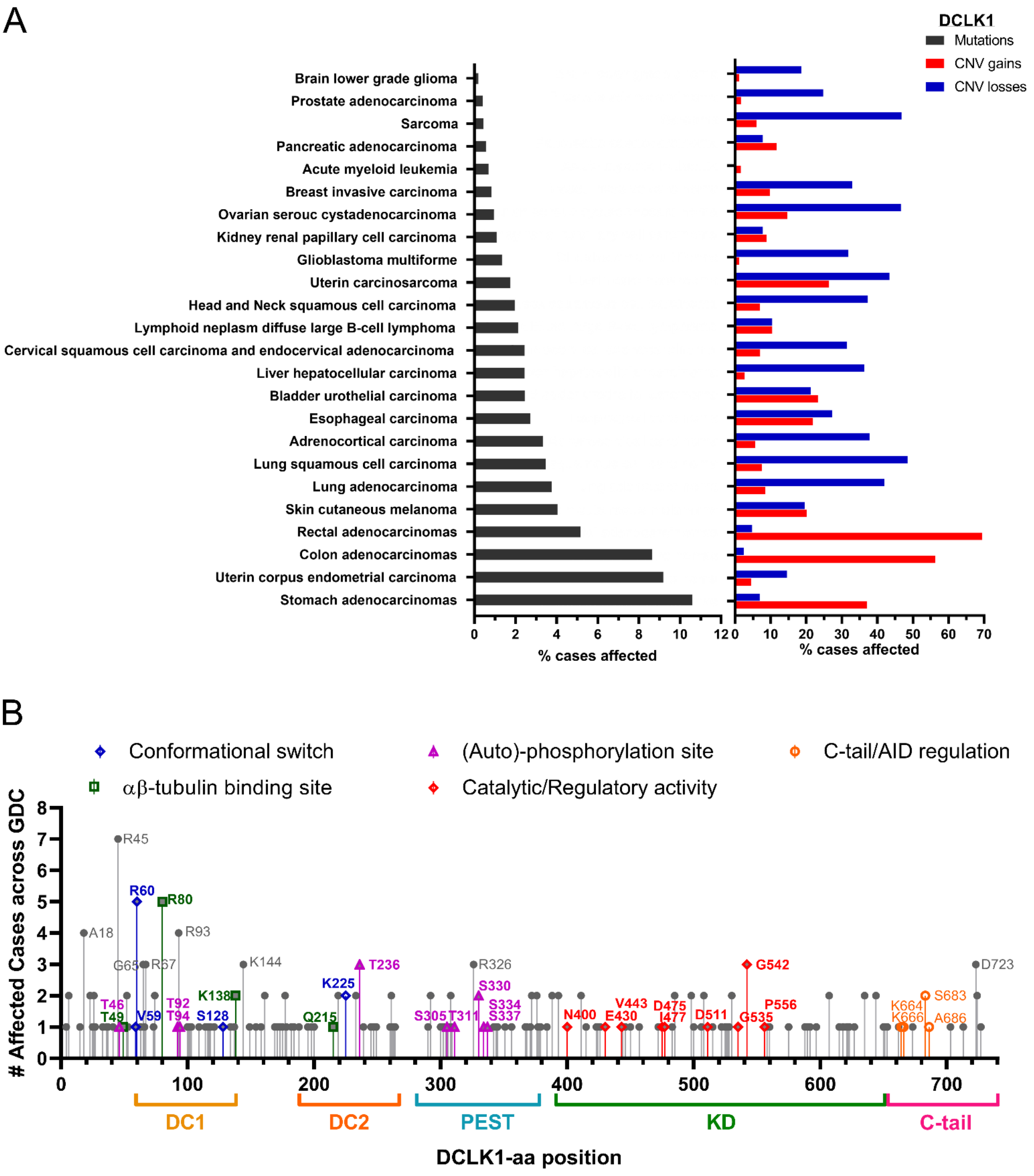

Figure S2 DCLK1 missense mutations in cancer. (**A**) The distribution of the 394 DCLK1 mutations and copy number variation (CNV) gains and losses identified in the genomic data commons (GDC) data-set for each malignancy. (**B**) Lollipop figure showing frequency and position of the DCLK1 missense mutations identified within the GDC cohort. Residues are only labelled if they coincide with a known functional residue, or with ≥ 3 incidences. With residues highlighted involved in the confirmational switch (blue), αβ-tubulin isomeric binding (green), (auto)-phosphorylation (purple), catalytic/regulatory kinase activity motif (red), and C-regulatory tail/auto-inhibitory domain (AID) (orange).

Table S1. Experimentally determined structures of the DC1 and DC2 domains from DCX and DCLK1, and the DCLK1 kinase domain.

Domain	Year	ID	Method	Res.	Protein-Isoform	Conformation	Notes	Reference
DC1	2020	6REV	Cryo-EM	3.8 Å	DCX	C-tail disordered	Cryo-EM structure bound to 13-protofilament GDP-microtubule	[73]
DC1	2020	6RFD	Cryo-EM	3.9 Å	DCX	C-tail disordered	Cryo-EM structure bound to 14-protofilament GDP-microtubule	[73]
DC1	2020	6RF8	Cryo-EM	3.8 Å	DCX	C-tail disordered	Cryo-EM structure bound to 13-protofilament GDP-microtubule	[136]
DC1	2016	5IKC	X-ray	2.06 Å	DCX	C-tail disordered	In complex with Fab 1/108	[75]
DC1	2016	5IN7	X-ray	2.48 Å	DCX	Closed	K215D/K216D mutant	[75]
DC1	2016	5IOI	X-ray	2.4 Å	DCX	Closed (chains C/F)<br>Open (chains A/E)<br>C-tail disordered (chains B/D)	K215D/K216D mutant	[75]
DC1	2016	5IO9	X-ray	1.3 Å	DCX	Closed	K215D/K216D mutant	[75]
DC1	2006	2BQQ	X-ray	2.2 Å	DCX	Open	K215D/K216D mutant	[85]
DC1	2003	1UF0	NMR		DCLK1	Closed		Unpublishe d
DC1	2003	1MG4	X-ray	1.504 Å	DCLK1	Closed	Unlabelled protein.	[80]
DC1	2003	1MFW	X-ray	1.6 Å	DCLK1	Closed	Selenomethionine labelled protein	[80]
DC1	2003	1MJD	NMR		DCX	Closed		[80]
DC2	2020	6RF2	Cryo-EM	4.2 Å	DCX	Monomeric	Cryo-EM structure bound to microtubules	[73]
DC2	2018	6FNZ	X-ray	2.23 Å	DCX	Domain-swapped dimer	Domain swapped dimer	[86]
DC2	2016	5IP4	X-ray	1.81 Å	DCX	Monomeric	In complex with nanobody XA4451. Closed conformation	[75]
KD	2021	7KX8	X-ray	3.1 Å	DCLK1-1/3<br>Residues 372-686	Active (Type 1)	DCLK1-Cter in complex with FMF-03-055-1	[54]
KD	2021	7KXW	X-ray	3.002 Å	DCLK1-1/2/3/4<br>Residues 372-649	Active (Type 1.5)	DCLK1-KD in complex with DCLK1-IN-1	[54]
KD	2021	7KX6	X-ray	2.5 Å	DCLK1-1/2/3/4<br>Residues 372-649	Active (Type 1)	DCLK1-KD in complex with XMD8-85	[54]
KD	2021	7F3G	X-ray	2.1 Å	DCLK1-1/2/3/4<br>Residues 372-649	Active (Type 1)	DCLK1 kinase domain in complex with ruxolitinib	[137]
KD	2021	6KYQ	X-ray	2.141 Å	DCLK1-2/4<br>Residues 379-703	Autoinhibited	DCLK1 Autoinhibited Kinase Domain	[51]
KD	2021	6KYR	X-ray	2.206 Å	DCLK1-2/4<br>Residues 379-703	Autoinhibited	DCLK1 mutant (P675L) Autoinhibited Kinase Domain	[51]
**Domain**	**Year**	**ID**	**Method**	**Res.**	**Protein-Isoform**	**Conformation**	**Notes**	**Reference**
KD	2016	5JZJ	X-ray	1.71 Å	DCLK1-1/2/3/4<br>Residues 372-649	Active (Type 1)	DCLK1-KD in complex with AMPPN	[53]
KD	2016	5JZN	X-ray	2.85 Å	DCLK1-1/2/3/4<br>Residues 372-649	Active (Type 1)	DCLK1-KD in complex with NVP-TAE684	[53]

Table S2. Functional residues of DCLK1 and their mutation frequency. The proposed function(s) for each identified site in DCX and matched or identified site in DCLK1 are given, including: its mutation frequency according to NCI's GDC portal [115], the amino acid substitutions observed; and the domain in DCLK1 in which it is located. The identified function of the residue which is color coded according to Figure 4 with (auto)phosphorylation sites (magenta), isomeric MT binding sites (green), conformational switch (blue), catalytic/regulatory kinase activity (other colors).

Identified DCX residue	Identified or matched DCLK1 residue	Mutation frequency	Substitution	Domain	Function	Reference
S28	S32				phosphorylation related actin/microtubule binding switch on DCX	[70]
					phosphorylated by CDK5 on DCX	[89]
	T46	1	T>M		(Auto)phosphorylation site identified in mass spectrometry based experiment	[52,53,83]
Q44	Q48				isomeric binding site of DC1 domain (of DCX) to αβ-tubulin	[73]
A45	T49	1	T>M	DC1	isomeric binding site of DC1 domain (of DCX) to αβ-tubulin	[73]
					(Auto)phosphorylation site identified in mass spectrometry based experiment	[83,52]
S47	S51			DC1	phospho-site identified with mass spectrometry based (altered upon C-tail deletion or T668A mutation)	[52]
					Phosphorylated by PKA and MARK on DCX	[20]
					Facilitates Kinesin-3 motor binding along microtubules	[74]
	S52	2	S>Y/C	DC1	Autophosphorylation site identified in mass spectrometry based experiment	[83]
A52	A56			DC1	isomeric binding site of DC1 domain (of DCX) to αβ-tubulin	[73]
K54	K58			DC1	Isomeric binding site of tryptophan -> open DC state	[75,85,80]
V55	V59	1	V>A	DC1	Isomeric binding site of tryptophan -> open DC state	[75,85,80]
R56	R60	5	R>C	DC1	Isomeric binding site of tryptophan -> open DC state	[75,85,80]
	S77			DC1	Phospho-site identified with mass spectrometry based (altered upon C-tail deletion or T668A mutation)	[52]
					DCLK1 phospho-site identified in mass spectrometry based experiment	[49]
R76	R80	5	R>W 3x<br>R>Q 2x	DC1	Isomeric binding site of DC1 domain (of DCX) to αβ-tubulin	[73]
R78	R82			DC1	Isomeric binding site of DC1 domain (of DCX) to αβ-tubulin	[73]
	S83			DC1	phospho-site identified with mass spectrometry based (altered upon C-tail deletion or T668A mutation)	[52]
D81	E85			DC1	Isomeric binding site of DC1 domain (of DCX) to αβ-tubulin	[73]
	T92	1	T>P	DC1	Autophosphorylation site identified in mass spectrometry based experiment	[52]
	T94	1	T>P	DC1	Autophosphorylation site identified in mass spectrometry based experiment	[52]

Identified DCX residue	Identified or matched DCLK1 residue	Mutation frequency	Substitution	Domain	Function	Reference
	S96			DC1	Phospho-site identified with mass spectrometry based (altered upon C-tail deletion or T668A mutation)	[52]
					Autophosphorylation site identified in mass spectrometry based experiment	[83]
N94	N98			DC1	Isomeric binding site of DC1 domain (of DCX) to αβ-tubulin	[73]
R102	R106			DC1	Isomeric binding site of DC1 domain (of DCX) to αβ-tubulin	[73]
	T107			DC1	Autophosphorylation site identified in mass spectrometry based experiment	[52]
	S119			DC1	phospho-site identified with mass spectrometry based (altered upon C-tail deletion or T668A mutation)	[52]
G122	G126			DC1	Isomeric binding site of tryptophan -> open DC state	[75,85,80]
E123	E127			DC1	Isomeric binding site of tryptophan -> open DC state	[75,85,80]
S124	S128	1	S>R	DC1	Isomeric binding site of tryptophan -> open DC state	[75,85,80]
				DC1	Phospho-site identified with mass spectrometry based (altered upon C-tail deletion or T668A mutation)	[52]
K134	K138	2	K>N	DC1	Isomeric binding site of DC1 domain (of DCX) to αβ-tubulin	[73]
	T143				Phospho-site identified with mass spectrometry based (altered upon C-tail deletion or T668A mutation)	[52]
W146	W150				Tryptophan responsible for the open/close confirmation of DC domains	[75,85,80]
	S151				Autophosphorylation site identified in mass spectrometry based experiment	[13,17,18]
	T156				Autophosphorylation site identified in mass spectrometry based experiment	[18]
	S158	1	S>W		Autophosphorylation site identified in mass spectrometry based experiment	[18]
	S160				Autophosphorylation site identified in mass spectrometry based experiment	[53,83,52]
	S164				Autophosphorylation site identified in mass spectrometry based experiment	[53,83,52]
	S165				Autophosphorylation site identified in mass spectrometry based experiment	[83]
	T168				Autophosphorylation site identified in mass spectrometry based experiment	[52]
	S172				Autophosphorylation site identified in mass spectrometry based experiment	[83]
	S174				Autophosphorylation site identified in mass spectrometry based experiment	[83]
	T189			DC2	Phospho-site identified with mass spectrometry based (altered upon C-tail deletion or T668A mutation)	[52]
	S193			DC2	Phospho-site identified with mass spectrometry based (altered upon C-tail deletion or T668A mutation)	[52]
N200	N206			DC2	Isomeric binding site of DC2 domains (of DCX) to αβ-tubulin	[73]
T203	T209			DC2	Isomeric binding site of DC2 domains (of DCX) to αβ-tubulin	[73]
H205	H211			DC2	Isomeric binding site of DC2 domains (of DCX) to αβ-tubulin	[73]

Identified DCX residue	Identified or matched DCLK1 residue	Mutation frequency	Substitution	Domain	Function	Reference
E208	E214			DC2	Isomeric binding site of DC2 domains (of DCX) to αβ-tubulin	[73]
Q209	Q215	1	Q>K	DC2	Isomeric binding site of DC2 domains (of DCX) to αβ-tubulin	[73]
	T218			DC2	Phospho-site identified with mass spectrometry based (altered upon C-tail deletion or T668A mutation)	[52]
K219	K225	1	K>T		KLET/KLDS hinge	[86]
L220	L226				KLET/KLDS hinge	[86]
E221	D227				KLET/KLDS hinge	[86]
T222	S228			DC2	KLET/KLDS hinge	[86]
					Isomeric binding site of DC2 domains (of DCX) to αβ-tubulin	[73]
					Phospho-site identified with mass spectrometry based (altered upon C-tail deletion or T668A mutation)	[52]
V224	V230			DC2	Isomeric binding site of DC2 domains (of DCX) to αβ-tubulin	[73]
	T236	3	T>A 1x<br>T>M 2x	DC2	Autophosphorylation site identified in mass spectrometry based experiment * Full length specific	[52]
	S274			PEST	Autophosphorylation site identified in mass spectrometry based experiment	[53,83]
S297	S298			PEST	Phospho-DCX by CDK5	[18]
	S305	1	S>G	PEST	DCLK1 phospho-site identified in mass spectrometry based experiment	[49]
	S307	1		PEST	DCLK1 phospho-site identified in mass spectrometry based experiment	[49]
					Autophosphorylation site identified in mass spectrometry based experiment	[53]
	T310			PEST	DCLK1 phospho-site identified in mass spectrometry based experiment	[49]
	T311	1	T>I	PEST	DCLK1 phospho-site identified in mass spectrometry based experiment	[49]
	S312			PEST	Autophosphorylation site identified in mass spectrometry based experiment	[53]
	S313			PEST	DCLK1 phospho-site identified in mass spectrometry based experiment	[49]
					Autophosphorylation site identified in mass spectrometry based experiment	[53]
	S320			PEST	Autophosphorylation site identified in mass spectrometry based experiment	[53]
T321	T324			PEST	Phospho-DCX by JNK	[105]
	S327			PEST	DCLK1 phospho-site identified in mass spectrometry based experiment	[49]
S332	S330	2	S>L	PEST	Phospho-DCX by JNK	[107]
					DCLK1 phospho-site identified in mass spectrometry based experiment	[49]
	S332			PEST	DCLK1 phospho-site identified in mass spectrometry based experiment	[49,48]
T331	S334	1	S>L	PEST	Phospho-DCX by JNK	[105]
					DCLK1 phospho-site identified in mass spectrometry based experiment	[49]

Identified DCX residue	Identified or matched DCLK1 residue	Mutation frequency	Substitution	Domain	Function	Reference
	T336			PEST	DCLK1 phospho-site identified in mass spectrometry based experiment	[49,48]
					Autophosphorylation site identified in mass spectrometry based experiment	[53]
S334	S337	1	S>N	PEST	Phospho-DCX by JNK	[105]
					DCLK1 phospho-site identified in mass spectrometry based experiment	[49,48]
	S340			PEST	DCLK1 phospho-site identified in mass spectrometry based experiment	[49]
	S364				DCLK1 phospho-site identified in mass spectrometry based experiment	[48]
	T395			KD	Autophosphorylation site identified in mass spectrometry based experiment	[83]
	N400	1	N>H	KD	Part of Glycine rich loop	[53]
	V404			KD	Critical residue in C-spine / ATP binding	[53]
	A417			KD	Critical residue in C-spine	[53]
	K419			KD	ATP binding	[53]
	E430	1	E>K	KD	Part of αC-helix	[53]
	E436			KD	ATP binding	[53]
	S438			KD	Autophosphorylation site identified in mass spectrometry based experiment	[83]
	L440			KD	Critical residue in R-spine	[53]
	V443	1	V>L	KD	Part of αC-helix	[53]
	L451			KD	Critical residue in R-spine	[53]
	L473			KD	Critical residue in C-spine	[53]
	D475	1	D>N	KD	Involved in hinge region with AID domain	[51]
	I477	1	I>T	KD	Involved in hinge region with AID domain	[51]
	M491			KD	Critical residue in C-spine	[53]
	H509			KD	HRD motif / Critical residue in R-spine	[53]
	R510			KD	HRD motif	[53]
	D511	1	D>N	KD	HRD motif / D511N is kinase dead mutant	[53]
	L517			KD	Critical residue in C-spine	[53]
	V519			KD	Critical residue in C-spine	[53]
	D533			KD	DFG motif	[53]
	F534			KD	DFG motif / Critical residue in R-spine	[53]
	G535	1	G>E	KD	DFG motif	[53]
	G542	3	G>V 1x	KD	part of the activation loop	[53]
			G>D 2x			
	T546			KD	Conserved phosphorylation-site in activation loop	[53]
	P556	1	P>A	KD	Part of the activation loop	[53]

Identified DCX residue	Identified or matched DCLK1 residue	Mutation frequency	Substitution	Domain	Function	Reference
	D569			KD	Critical residue in R-spine	[53]
	I576			KD	Critical residue in C-spine	[53]
	L580			KD	Critical residue in C-spine	[53]
	S637			KD	Autophosphorylation site identified in mass spectrometry based experiment	[53]
	V661			C-tail	Hydrophobic interaction of R1 with KD	[51]
	K664	1	K>N	C-tail	Part of R1 helix	[51]
	I665			C-tail	Hydrophobic interaction of R1 with KD	[51]
	K666	1	K>N	C-tail	Part of R1 helix	[51]
	T668			C-tail	Autophosphorylation-site for AID domain regulation	[52]
	F669			C-tail	Hydrophobic interaction of R1 with KD	[51]
	T678*			C-tail	Autophosphorylation site identified in mass spectrometry based experiment	[53]
	V682			C-tail	Hydrophobic interaction of R2 with KD	[51]
	S683	2	S>Y	C-tail	Part of R2 helix	[51]
	V684			C-tail	Hydrophobic interaction of R2 with KD	[51]
	I685			C-tail	Hydrophobic interaction of R2 with KD	[51]
	A686	1	A>T	C-tail	Part of R2 helix	[51]
	K692			C-tail	Key residue of R3 that forms salt bridges with D511 and D533	[51]
	T692*			C-tail	Autophosphorylation site identified in mass spectrometry based experiment	[53]
	R698			C-tail	Hydrophilic interaction with KD	[51]
	R701			C-tail	Hydrophilic interaction with KD	[51]
	S720			C-tail	DCLK1 phospho-site identified in mass spectrometry based experiment	[48]
	S726			C-tail	DCLK1 phospho-site identified in mass spectrometry based experiment	[48]

Table S3. Predicted impact of point mutations on the structural stability of DCLK1. Predictions were performed using the DynaMut2 server [138] on mutations for which an experimental structure was available (either of DCX or DCLK1). The impact on stability was classified according to the change in Gibbs Free Energy ($\Delta\Delta G^{Stability}$): neutral ($-0.5 < \Delta\Delta G^{Stability} < 0.5$ kcal/mol), mildly destabilising ($0.5 \leq \Delta\Delta G^{Stability} < 1$ kcal/mol), or destabilising ($\Delta\Delta G^{Stability} \geq 1$ kcal/mol).

**Mutation**	**Domain**	**PDB ID (Protein)**	**Predicted stability change ($\Delta\Delta G^{Stability}$) in kcal/mol**	**Predicted Impact**
T49M	N-terminal region	6REV (DCX-Tubulin, A45M)	-0.7	Mildly destabilising
V59A	DC1	1MG4 (DCLK1)	-2.41	Destabilising
R60C	DC1	1MG4 (DCLK1)	-1.78	Destabilising
G65A	DC1	6REV (DCX-Tubulin, G61A)	-0.21	Neutral
G65R	DC1	6REV (DCX-Tubulin, G61R)	-0.62	Mildly destabilising
R67G	DC1	1MG4 (DCLK1)	-0.49	Mildly destabilising
R67Q	DC1	1MG4 (DCLK1)	-0.38	Neutral
R80Q	DC1	6REV (DCX-Tubulin, R76Q)	-0.54	Mildly destabilising
R80W	DC1	6REV (DCX-Tubulin, R76W)	-1.02	Destabilising
T92P	DC1	6REV (DCX-Tubulin, T88P)	-0.39	Neutral
R93Q	DC1	6REV (DCX-Tubulin, R89N)	-0.54	Mildly destabilising
T94P	DC1	6REV (DCX-Tubulin, S90P)	-0.44	Neutral
S128R	DC1	1MG4 (DCLK1)	0.42	Neutral
K138N	DC1	6REV (DCX-Tubulin, K134N)	-0.31	Neutral
K144N	DC1	1MG4 (DCLK1)	0.43	Neutral
K144R	DC1	1MG4 (DCLK1)	0.1	Neutral
Q215K	DC2	6RF2 (DCX-Tubulin, Q209K)	0.23	Neutral
K225R	DC2	6RF2 (DCX-Tubulin, K219R)	-0.07	Neutral
K225T	DC2	6RF2 (DCX-Tubulin, K219R)	-0.38	Neutral
T236A	DC2	5IP4 (DCX, T230A)	-0.81	Mildly destabilising
T236M	DC2	5IP4 (DCX, T230M)	0.1	Neutral
N400H	Kinase domain	5JZJ (DCLK1)	-0.07	Neutral
E430K	Kinase domain	5JZJ (DCLK1)	0.31	Neutral
V443L	Kinase domain	5JZJ (DCLK1)	-0.5	Mildly destabilising
D475N	Kinase domain	5JZJ (DCLK1)	0.05	Neutral
I477T	Kinase domain	5JZJ (DCLK1)	-2.82	Destabilising
D511N	Kinase domain	5JZJ (DCLK1)	-0.37	Neutral
G535E	Kinase domain	5JZJ (DCLK1)	-1.68	Destabilising
G542D	Kinase domain	5JZJ (DCLK1)	-0.27	Neutral
G542V	Kinase domain	5JZJ (DCLK1)	-1.33	Destabilising
P556A	Kinase domain	5JZJ (DCLK1)	-1.08	Destabilising
K664N	C-tail	6KYQ (DCLK1)	-1.54	Destabilising
K666N	C-tail	6KYQ (DCLK1)	-0.08	Neutral
P673H	C-tail	6KYQ (DCLK1)	-0.11	Neutral
S683Y	C-tail	6KYQ (DCLK1)	-0.99	Destabilising
A686T	C-tail	6KYQ (DCLK1)	-0.78	Mildly destabilising

References

18. Tanaka, T.; Serneo, F.F.; Tseng, H.C.; Kulkarni, A.B.; Tsai, L.H.; Gleeson, J.G. Cdk5 Phosphorylation of Doublecortin Ser297 Regulates Its Effect on Neuronal Migration. *Neuron* **2004**, *41*, 215–227. https://doi.org/10.1016/S0896-6273(03)00852-3.
20. Schaar, B.T.; Kinoshita, K.; McConnell, S.K. Doublecortin Microtubule Affinity Is Regulated by a Balance of Kinase and Phosphatase Activity at the Leading Edge of Migrating Neurons. *Neuron* **2004**, *41*, 203–213. https://doi.org/10.1016/s0896-6273(03)00843-2.
48. Ferguson, F.M.; Nabet, B.; Raghavan, S.; Liu, Y.; Leggett, A.L.; Kuljanin, M.; Kalekar, R.L.; Yang, A.; He, S.; Wang, J.; et al. Discovery of a Selective Inhibitor of Doublecortin like Kinase 1. *Nat. Chem. Biol.* **2020**, *16*, 635–643. https://doi.org/10.1038/s41589-020-0506-0.
49. Liu, Y.; Ferguson, F.M.; Li, L.; Kuljanin, M.; Mills, C.E.; Subramanian, K.; Harshbarger, W.; Gondi, S.; Wang, J.; Sorger, P.K.; et al. Chemical Biology Toolkit for DCLK1 Reveals Connection to RNA Processing. *Cell Chem. Biol.* **2020**, *27*, 1229–1240.e4. https://doi.org/10.1016/j.chembiol.2020.07.011.
51. Cheng, L.; Yang, Z.; Guo, W.; Wu, C.; Liang, S.; Tong, A.; Cao, Z.; Thorne, R.F.; Yang, S.-Y.; Yu, Y.; et al. DCLK1 Autoinhibition and Activation in Tumorigenesis. *Innovation* **2022**, *3*, 100191. https://doi.org/10.1016/j.xinn.2021.100191.
52. Agulto, R.L.; Rogers, M.M.; Tan, T.C.; Ramkumar, A.; Downing, A.M.; Bodin, H.; Castro, J.; Nowakowski, D.W.; Ori-McKenney, K.M. Autoregulatory Control of Microtubule Binding in Doublecortin-like Kinase 1. *eLife* **2021**, *10*, e60126. https://doi.org/10.7554/eLife.60126.
53. Patel, O.; Dai, W.; Mentzel, M.; Griffin, M.D.W.; Serindoux, J.; Gay, Y.; Fischer, S.; Sterle, S.; Kropp, A.; Burns, C.J.; et al. Biochemical and Structural Insights into Doublecortin-like Kinase Domain 1. *Structure* **2016**, *24*, 1550–1531. https://doi.org/10.1016/j.str.2016.07.008.
54. Patel, O.; Roy, M.J.; Kropp, A.; Hardy, J.M.; Dai, W.; Lucet, I.S. Structural Basis for Small Molecule Targeting of Doublecortin Like Kinase 1 with DCLK1-IN-1. *Commun. Biol.* **2021**, *4*, 1105. https://doi.org/10.1038/s42003-021-02631-y.
70. Moslehi, M.; Ng, D.C.H.; Bogoyevitch, M.A. Doublecortin X (DCX) Serine 28 Phosphorylation Is a Regulatory Switch, Modulating Association of DCX with Microtubules and Actin Filaments. *Biochim. Biophys. Acta—Mol. Cell Res.* **2019**, *1866*, 638–649. https://doi.org/10.1016/j.bbamcr.2019.01.003.
73. Manka, S.W.; Moores, C.A. Pseudo-repeats in Doublecortin Make Distinct Mechanistic Contributions to Microtubule Regulation. *EMBO Rep.* **2020**, *21*, e51534. https://doi.org/10.15252/embr.202051534.
74. Liu, J.S.; Schubert, C.R.; Fu, X.; Fourniol, F.J.; Jaiswal, J.K.; Houdusse, A.; Stultz, C.M.; Moores, C.A.; Walsh, C.A. Molecular Basis for Specific Regulation of Neuronal Kinesin-3 Motors by Doublecortin Family Proteins. *Mol. Cell* **2012**, *47*, 707–721. https://doi.org/10.1016/j.molcel.2012.06.025.
75. Burger, D.; Stihle, M.; Sharma, A.; Di Lello, P.; Benz, J.; D'Arcy, B.; Debulpaep, M.; Fry, D.; Huber, W.; Kremer, T.; et al. Crystal Structures of the Human Doublecortin C- and N-Terminal Domains in Complex with Specific Antibodies. *J. Biol. Chem.* **2016**, *291*, 16292–16306, doi:10.1074/jbc.M116.726547.
80. Kim, M.H.; Cierpicki, T.; Derewenda, U.; Krowarsch, D.; Feng, Y.; Devedjiev, Y.; Dauter, Z.; Walsh, C.A.; Otlewski, J.; Bushweller, J.H.; et al. The DCX-Domain Tandems of Doublecortin and Doublecortin-like Kinase. *Nat. Struct. Biol.* **2003**, *10*, 324–333. https://doi.org/10.1038/nsb918.
83. Sugiyama, N.; Imamura, H.; Ishihama, Y. Large-Scale Discovery of Substrates of the Human Kinome. *Sci. Rep.* **2019**, *9*, 10503. https://doi.org/10.1038/s41598-019-46385-4.
85. Cierpicki, T.; Kim, M.H.; Cooper, D.R.; Derewenda, U.; Bushweller, J.H.; Derewenda, Z.S. The DC-Module of Doublecortin: Dynamics, Domain Boundaries, and Functional Implications. *Proteins Struct. Funct. Bioinform.* **2006**, *64*, 874–882. https://doi.org/10.1002/prot.21068.
86. Rufer, A.C.; Kusznir, E.; Burger, D.; Stihle, M.; Ruf, A.; Rudolph, M.G. Domain Swap in the C-Terminal Ubiquitin-like Domain of Human Doublecortin. *Acta Crystallogr. Sect. D Struct. Biol.* **2018**, *74*, 450–462. https://doi.org/10.1107/S2059798318004813.
89. Graham, M.E.; Ruma-Haynes, P.; Capes-Davis, A.G.; Dunn, J.M.; Tan, T.C.; Valova, V.A.; Robinson, P.J.; Jeffrey, P.L. Multisite Phosphorylation of Doublecortin by Cyclin-Dependent Kinase 5. *Biochem. J.* **2004**, *381*, 471–481. https://doi.org/10.1042/BJ20040324.
95. Obenauer, J.C.; Cantley, L.C.; Yaffe, M.B. Scansite 2.0: Proteome-Wide Prediction of Cell Signaling Interactions Using Short Sequence Motifs. *Nucleic Acids Res.* **2003**, *31*, 3635–3641. https://doi.org/10.1093/nar/gkg584.
105. Gdalyahu, A.; Ghosh, I.; Levy, T.; Sapir, T.; Sapoznik, S.; Fishler, Y.; Azoulai, D.; Reiner, O. DCX, a New Mediator of the JNK Pathway. *EMBO J.* **2004**, *23*, 823–832. https://doi.org/10.1038/sj.emboj.7600079.
107. Jin, J.; Suzuki, H.; Hirai, S.; Mikoshiba, K.; Ohshima, T. JNK Phosphorylates Ser332 of Doublecortin and Regulates Its Function in Neurite Extension and Neuronal Migration. *Dev. Neurobiol.* **2010**, *70*, 929–942. https://doi.org/10.1002/dneu.20833.
115. Grossman, R.L.; Heath, A.P.; Ferretti, V.; Varmus, H.E.; Lowy, D.R.; Kibbe, W.A.; Staudt, L.M. Toward a Shared Vision for Cancer Genomic Data. *N. Engl. J. Med.* **2016**, *375*, 1109–1112. https://doi.org/10.1056/NEJMp1607591.
120. Liu, Z.-X.; Yu, K.; Dong, J.; Zhao, L.; Liu, Z.; Zhang, Q.; Li, S.; Du, Y.; Cheng, H. Precise Prediction of Calpain Cleavage Sites and Their Aberrance Caused by Mutations in Cancer. *Front. Genet.* **2019**, *10*, 715. https://doi.org/10.3389/fgene.2019.00715.
135. Kumar, M.; Michael, S.; Alvarado-Valverde, J.; Mészáros, B.; Sámano-Sánchez, H.; Zeke, A.; Dobson, L.; Lazar, T.; Örd, M.; Nagpal, A.; et al. The Eukaryotic Linear Motif Resource: 2022 Release. *Nucleic Acids Res.* **2022**, *50*, D497–D508, doi:10.1093/nar/gkab975.
136. Cook, A.D.; Manka, S.W.; Wang, S.; Moores, C.A.; Atherton, J. A Microtubule RELION-Based Pipeline for Cryo-EM Image Processing. *J. Struct. Biol.* **2020**, *209*, 107402, doi:10.1016/j.jsb.2019.10.004.
137. Jang, D.M.; Lim, H.J.; Hahn, H.; Lee, Y.; Kim, H.K.; Kim, H.S. Structural Basis of Inhibition of DCLK1 by Ruxolitinib. *Int. J. Mol. Sci.* **2021**, *22*, 8488, doi:10.3390/ijms22168488.
138. Rodrigues, C.H.M.; Pires, D.E.V.; Ascher, D.B. DynaMut2: Assessing Changes in Stability and Flexibility upon Single and Multiple Point Missense Mutations. *Protein Sci.* **2021**, *30*, 60–69, doi:10.1002/pro.3942.
